# Metastasis of a cecal adenocarcinoma to the prostate five years after a right hemicolectomy: a case report

**DOI:** 10.1186/1752-1947-5-223

**Published:** 2011-06-21

**Authors:** Fady R Youssef, Leanne Hunt, Pieter D Meiring, Danesh R Taraporewalla, Robin Gupta, Mike J James

**Affiliations:** 1 Royal Hallamshire Hospital, Glossop Road, Sheffield, S10 2JF, UK; 2 Chesterfield Royal Hospital, Chesterfield Road, Calow, Chesterfield, S44 5BL, UK

## Abstract

**Introduction:**

Prostatic metastasis from a primary bowel adenocarcinoma has been only rarely reported in the medical literature. The case reported here is rare in the fact that the primary tumor was from a right-sided bowel adenocarcinoma. It is unusual because initial immunostaining was not fully conclusive, and so a relatively new method of immunostaining, CDX2, was used to ascertain its histopathology.

**Case presentation:**

We describe the case of a 54-year-old Caucasian man who had a right hemicolectomy for a primary cecal adenocarcinoma, which was completely excised. Following the procedure, he received adjuvant chemotherapy. Computed tomography scans showed no evidence of local recurrence or metastatic disease. Then, five years later, he presented to his general practitioner with urinary symptoms. An abnormal prostate was palpated on digital rectal examination. Trans-rectal prostatic biopsies were performed, which showed colorectal metastases within the prostate gland. This was confirmed with CDX2 immunohistochemistry. There was no further evidence of distant metastases on positron emission tomography-computed tomography scans.

**Conclusions:**

This case demonstrates a rare isolated hematogenous spread to the prostate from a primary cecal adenocarcinoma, several years after definitive treatment and excision. This highlights the importance of accurate immunohistochemistry and imaging in planning further management and treatment.

## Introduction

Prostatic metastasis from a primary bowel adenocarcinoma has been only rarely reported in the medical literature. We describe a case of a rare metastasis from a right-sided primary bowel adenocarcinoma to the prostate gland. It is unusual because a relatively new method of immunostaining, CDX2, was used to ascertain its histopathology.

## Case presentation

A 54-year-old Caucasian man was admitted to our hospital with an acute history of abdominal pain and vomiting, and a four-month history of changes in bowel habits. A computed tomography (CT) scan of his abdomen and pelvis confirmed a small bowel obstruction secondary to a mass lesion at the cecal pole. A few lymph nodes were identified adjacent to the cecal pole, measuring approximately 1 cm in size.

On laparotomy, a mobile cecal tumor was found, with no other evidence of intra-abdominal metastatic disease. A right hemicolectomy was performed. He made an uneventful recovery and was discharged one week after surgery.

Histology results showed a moderately differentiated mucinous adenocarcinoma (pT4, N2, Mx) Dukes C1, with an incidental carcinoid tumor in the appendix. Surgical resection margins were clear. Carcinogenic embryonic antigen (CEA) staining was diffusely positive, cytokeratin 20 (CK20) staining was focally positive, and cytokeratin 7 (CK7) staining was negative. A CK7-negative and CK20-positive profile favors a primary colorectal tumor, although CK20 staining was weakly positive.

He underwent adjuvant chemotherapy as part of the QUASAR ('Quick and Simple and Reliable') trial [1]. A post-operative CT scan at six months demonstrated two small liver lesions and a small lung lesion. These had not changed on repeat scans at three months and 12 months. All further follow-up consultations and investigations showed no evidence of recurrence.

After five years of follow-up, he was referred by his general practitioner to our urology department with lower urinary tract symptoms and an abnormal prostate on digital rectal examination. There was a large, suspected to be malignant, extrinsic pelvic mass that could not be palpated separately from the prostate. His prostate specific antigen (PSA) level was 1.3 ng/ml (normal range 0 to 4 ng/ml).

Trans-rectal ultrasound prostate biopsies were taken and results showed normal background prostatic tissue, with all cores infiltrated with mucinous adenocarcinoma. Immunohistochemistry results were positive for CEA staining but negative for PSA, CK7 and CK20 staining.

**Figure 1:**
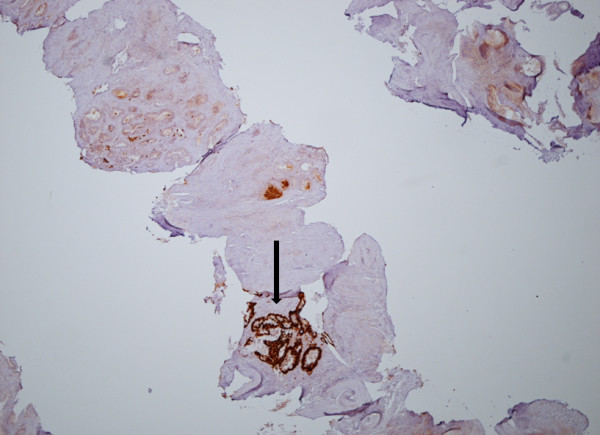
**Prostatic core demonstrating mucinous adenocarcinoma with positive CDX2 staining**.

The original bowel specimen was again analyzed and the morphology of the tumor in the bowel specimen was identical to that in the prostate. Additional staining was performed, and both the bowel and prostatic specimens tested positive for CDX2 (Figure 1). It was concluded that the prostate biopsies contained mucin-secreting adenocarcinoma with intestinal differentiation, as indicated by the presence of CDX2, morphologically identical to the original primary bowel carcinoma, thus representing a metastasis of this tumor.

**Figure 2:**
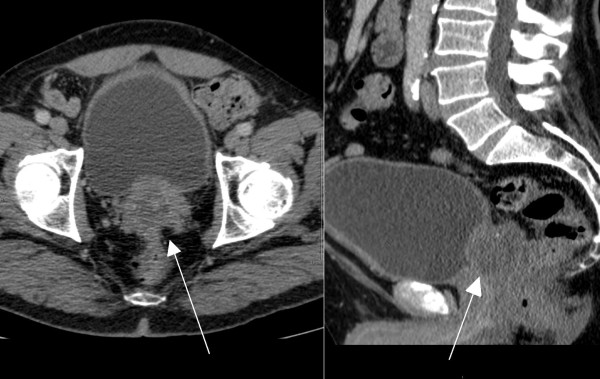
**Computed tomography (CT) images demonstrating the prostate with metastatic invasion**.

**Figure 3:**
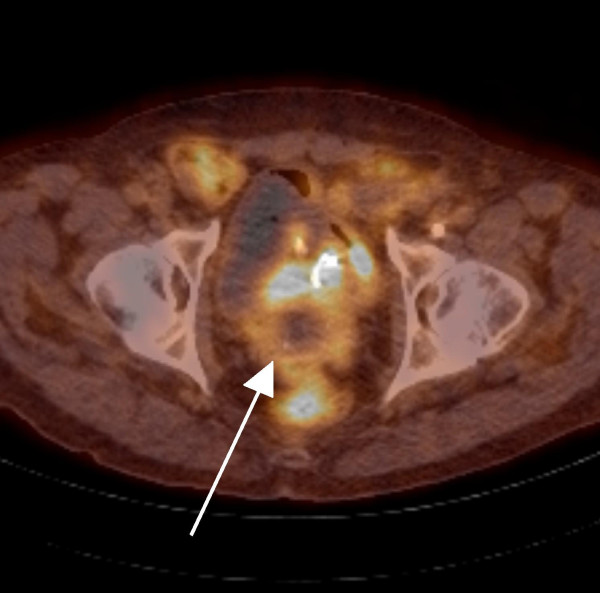
**Fused positron emission tomography-computed tomography (PET-CT) cross-sectional image demonstrating prostatic metastasis with central necrosis**.

A fused positron emission tomography-computed tomography (PET-CT) scan was performed to exclude further distant metastatic disease. This demonstrated increased uptake in the prostate with central necrosis, consistent with a metastasis (Figures 2 and 3). There were no other signs of metastatic disease elsewhere. Our patient is now being considered for radical pelvic exenteration with curative intent. Other treatment modalities would not offer this.

## Discussion

There have only rarely been other reported cases of prostatic metastasis from primary bowel adenocarcinomas [2-4]. Other primary malignancies have reportedly metastasized to the prostate. These include malignant melanoma, lung, pancreas, stomach, penis and larynx [5].

CDX2 is a monoclonal antibody to the intestinal-epithelia-specific nuclear transcription factor, and is a relatively new marker for gastrointestinal tumors. It is expressed in the nuclei of intestinal cells throughout the intestine from duodenum to rectum [6]. Immunohistochemistry has demonstrated 60% to 98% CDX2 expression in primary and secondary colorectal adenocarcinomas [7,8]. CDX2 is also expressed in tumors from other gastrointestinal sites, including the esophagus, stomach, pancreatobiliary, gastrointestinal carcinoids and liver. High levels have also been detected in mucinous ovarian carcinomas and adenocarcinomas originating from the urinary bladder [9].

Owens *et al*. demonstrated 60% positivity for CDX2 in primary colorectal cancers versus 0% expression in primary prostate cancers [7].

CDX2's positive nature in the prostate specimens, as well as the original bowel specimens from this case, strongly correlate with a diagnosis of metastatic spread from the original cecal adenocarcinoma to the prostate. One would expect CK20 staining to be positive in the prostatic specimens, but staining on the original bowel tumor was only weakly positive, which may account for the metastatic focus showing a negative CK20 profile. This stresses the importance of advanced immunohistochemical and pathological techniques in differentiating tumor origin in patients with previous malignancy and uncommon sites of metastatic disease. This enables accurate diagnosis and appropriate treatment.

## Conclusions

This case demonstrates a rare isolated hematogenous spread to the prostate from a primary cecal adenocarcinoma, several years after definitive treatment and excision.

## Consent

Written informed consent was obtained from the patient for publication of this case report and any accompanying images. A copy of the written consent is available for review by the Editor-in-Chief of this journal.

## Competing interests

The authors declare that they have no competing interests.

## Authors' contributions

FRY drafted and wrote the manuscript and summarized pertinent features of the case. LH aided in the summary of the case as a whole and was a major contributor to writing the manuscript. PDM selected and annotated appropriate images from cross-sectional imaging. DRT selected and annotated appropriate images from histopathology slides and reviewed all available histology to ensure an accurate diagnosis was made. RG aided in the summary of the case from a colorectal perspective. MJJ aided in the summary of the case from a urological perspective. All authors read and approved the final manuscript
